# When is proton pump inhibitor use appropriate?

**DOI:** 10.1186/s12916-017-0804-x

**Published:** 2017-02-21

**Authors:** Rena Yadlapati, Peter J. Kahrilas

**Affiliations:** 1 Department of Medicine, The Feinberg School of Medicine, Northwestern University, Chicago, IL USA; 2 Division of Gastroenterology and Hepatology, Department of Medicine, Northwestern University, Feinberg School of Medicine, 676 N. St. Clair Street, 14th Floor, Chicago, IL 60611 USA

**Keywords:** Proton pump inhibitors, Gastroesophageal reflux disease, Indication

## Abstract

Proton pump inhibitor (PPI) therapy is commonly used outside of Food and Drug Administration indication for a broad range of conditions such as extra-esophageal reflux and PPI-responsive esophageal eosinophilia. While this may be appropriate in some scenarios, it has also resulted in widespread inappropriate PPI use. At the same time, data suggesting adverse effects of long-term PPI therapy are multiplying, albeit mainly from low quality studies. The systematic review by Scarpignato et al. (*BMC Med* 14:179, 2016) addresses this dilemma with a comprehensive analysis of the risks and benefits of PPI use. The authors concluded that, while PPIs are highly efficacious in erosive acid-peptic disorders, efficacy is not equaled in other conditions. In some instances, they found no supportive evidence of benefit. With respect to side effects, they indicated that the questionable harms associated with PPI therapy do not outweigh the benefits afforded by appropriate PPI use. However, inappropriate PPI use results in increased healthcare costs and unnecessary exposure to potential adverse effects. Ideally, PPI therapy should be personalized, based on indication, effectiveness, patient preference, and risk assessment.

Please see related article: http://bmcmedicine.biomedcentral.com/articles/10.1186/s12916-016-0718-z.

## Background

Proton pump inhibitors (PPIs) have revolutionized the medical approach to upper gastrointestinal disorders. Initially developed as a treatment for reflux esophagitis, these potent inhibitors of gastric acid secretion have subsequently proven effective for a broad range of syndromes known (or suspected) to be attributable to acid reflux, acid secretion, or acid hypersecretion. Combined with seemingly excellent safety and tolerance, these broadened indications triggered an exponential increase in PPI use (and consequently cost) worldwide [1, 2]. Furthermore, PPIs have become a victim of their own success. Not only are they now often prescribed for syndromes of dubious merit (“silent reflux”), but an almost cultish faith in potent acid suppression as a treatment for all that ails the human race has led to progressive escalation of PPI dosage and potency. Indeed, a vocabulary has emerged around the concept of “PPI failure”, often ignoring the possibility that the condition in question had no relationship to gastric acid secretion in the first place. Consequently, it is no surprise that, in less than 30 years, PPIs have evolved from wonder drugs to a major healthcare epidemic. Coupled with this has been unprecedented scrutiny of the safety of chronic PPI use; it is in this background that Scarpignato et al. [3] author a position paper on “Effective and safe proton pump inhibitor therapy in acid-related diseases”.

## Use of PPIs

Coincident with PPI usage, the literature surrounding PPI safety and efficacy is growing exponentially, making it difficult to delineate an evidence-based guideline on their use. In the study by Scarpignato et al. [3], committees on behalf of three Italian scientific societies collaborated with impressive panels of expert international reviewers to address 13 clinical scenarios in which uncertainty exists about how to prescribe PPIs and where drug misuse is common. They performed a systematic literature review inclusive of almost 500 papers and present a narrative review of the safety and appropriateness of PPI therapy in each scenario. Table 1 summarizes the key messages regarding appropriate PPI use in the 13 scenarios, organized into those appropriate for long-term or short-term PPI use, or not at all. Examining this compendium, one is immediately struck by the fact that the table includes many usages for which there are no Food and Drug Administration (FDA) indications. Apparently, the world literature provides ample evidence for the efficacy of PPIs outside of FDA indications.

**Table 1:** Summary of the conclusions by Scarpignato et al. [3] regarding the appropriateness of proton pump inhibitor (PPI) therapy in 13 clinical scenarios of uncertainty and common misuse

	Reason for use
Long-term PPI therapy appropriate	• Barrett’s esophagus • Healing and maintenance of healed Los Angeles grade C or D erosive esophagitis^a^ • PPI-responsive esophageal eosinophilia • Idiopathic (*H. pylori* and NSAID/aspirin negative) peptic ulcer disease • Zollinger–Ellison disease^a,b^ • PPI-responsive GERD/non-erosive reflux disease^a,c^ • Long-term non-selective NSAID users at high-risk for upper GI complications or long-term cox-2 inhibitor users with a prior episode of GI bleeding^a^ • Anti-platelet therapy in patients at high-risk for upper GI complications (age > 65 years or concomitant use of corticosteroids or anticoagulants or history of peptic ulcer disease) • Steatorrhea refractory to enzyme replacement therapy in chronic pancreatitis
Short-term PPI therapy appropriate (4- to 12-week course)	• Healing of Los Angeles grade A or B erosive esophagitis^a^ • Eosinophilic esophagitis • *H. pylori* eradication (in combination with antibiotics)^a,d^ • Stress ulcer prophylaxis in high-risk patients (i.e., critically ill patients with respiratory failure or coagulopathy) • Functional dyspepsia • Treatment and maintenance of peptic ulcer disease^a^ • Prior to endoscopy for acute upper GI bleeding • Following endoscopic treatment of a high-risk ulcer GI bleed
PPI use not appropriate	• Corticosteroid users without concomitant NSAID therapy • To prevent bleeding from hypertensive gastropathy in cirrhotic patients • Acute pancreatitis • Stress ulcer prophylaxis in non-critically ill hospitalized patients that are not at high-risk for ulcer formation and GI bleeding
PPI use of uncertain benefit	• PPI non-responsive GERD • Extra-digestive GERD

^a^FDA approved indications

^b^Requires 3–4 times the usual dose (PPI therapy is typically started as single dose)

^c^In these cases, a PPI taper should be attempted to the lowest effective dose, on demand dosing, or intermittent dosing

^d^In this case, a 1 to 2 week course of PPI therapy for *H. pylori* eradication in conjunction with antibiotics is appropriate

*GERD* gastroesophageal reflux disease, *GI* gastrointestinal, *NSAID* non-steroidal anti-inflammatory drugs

For the current FDA indications for PPI use (indicated in Table 1, with some liberties taken) there is little reason to quibble about their appropriateness. It is when the recommendations go beyond FDA recommendations and in the gray areas where there is great potential over-use (and conversely, over-regulation), these include eosinophilic esophagitis, non-erosive reflux disease, chemoprevention in Barrett’s esophagus, dyspepsia, and extra-esophageal reflux. Not surprisingly, the evidence supporting PPI use in these indications is generally weak. However, saying that the evidence is weak is not the same as stating that it should not be done. Rather, it becomes incumbent on the practitioner to establish the effectiveness of the PPI, or the out-of-indication PPI dose, for the particular patient. In the case of eosinophilic esophagitis, long-term PPI use requires endoscopic/histopathologic verification that PPI use cleared mucosal eosinophilia. In the case of non-erosive reflux disease it means coupling PPI therapy with weight management and lifestyle modification with the ultimate goal of tapering PPI therapy to the lowest effective dose. Prior relevant studies have shown that PPI dosage can be successfully reduced in the majority of such patients and entirely discontinued in nearly 20% [2].

The role of PPI therapy for managing extra-esophageal symptoms that are potentially from gastroesophageal reflux is even more perplexing. Scarpignato et al. [3] highlight the paucity of high-quality data regarding PPI therapy for non-cardiac chest pain, laryngeal complaints, asthma, dental erosions, and chronic cough. Paradoxically, this has led to the widespread practice of treating these conditions with high doses of PPIs for extended periods of time. Furthermore, with no specified alternative management strategy, this frequently leads to long-term high-dose PPI usage in these conditions regardless of effectiveness [4, 5]. More than any, these patients benefit from further physiological investigation (manometry, reflux monitoring) to either implicate PPI-refractory GERD as a cause of their symptoms or to justify PPI discontinuation. Based on personal experience, the appropriate intervention is often PPI discontinuation in patients with isolated extra-esophageal symptoms that have not responded to a PPI trial. Continuing PPI therapy in such circumstances provides no benefit and simply exposes patients to the risk of therapy, the other focus to the Scarpignato review [3].

The list of safety concerns related to long-term PPI use is growing, both in number and in public visibility. Although largely based on low quality data and, in many instances, refuted by higher quality data, the warnings are out there on the TV, the internet, and the package inserts. This has resulted in widespread PPI angst among patients and not a day goes by that this is not the topic of a patient consultation in a specialty gastrointestinal disorders practice. Scarpignato et al. [3] comprehensively review both the digestive and extra-digestive concerns about PPI usage, but ultimately reiterate that PPIs are well tolerated and that the benefits of PPI treatment outweigh potential risks when PPIs are used for an appropriate indication. Table 2 summarizes available safety information on long-term PPI use with the concerns grouped by the strength of substantiating data and significance. Evident in the table, there is little there of sufficient concern to alter practice, providing that PPI use is appropriate.

**Table 2:** Quality of evidence and risks of adverse effects associated with long-term proton pump inhibitors (PPIs)

	Potential adverse effect	Nature of evidence	Risk estimate
Causality established, idiosyncratic, rare	Acute interstitial nephritis	Observational, case–control	OR 5.16 (2.21–12.05)
Causality proven but of minimal significance	Fundic gland polyps	Observational	OR 2.2 (1.3–3.8) [6]
	B12 deficiency	Observational, case–control	OR 1.65 (1.58–1.73) [7]
Weak association, causality probable	Small intestinal bacterial overgrowth	Meta-analysis	OR 2.28 (1.23–4.21) [8]
	Spontaneous bacterial peritonitis in cirrhotic patients	Systematic review/meta-analysis	OR 2.17 (1.46–3.23) [9]
	Hepatic encephalopathy in cirrhotic patients	Observational, case–control	Dose dependent response, up to OR 3.01 (1.78–5.10) [10]
	*Clostridium difficile* infection	Observational cohort study	OR 2.10 (1.20–3.50)
	Iron deficiency	Observational, case control	OR 2.49 (2.35–2.64) [11]
	Hypomagnesemia	Observational, population-based cohort	OR 2.00 (1.36–2.93)^a^ [12]
Weak association, unproven causality	Bone fracture	Observational, case–control	OR 2.65 (1.80–3.90)
	Chronic kidney disease	Observational, population-based cohort	HR 1.50 (1.14–1.96) [13]
	Dementia	Prospective observational cohort	HR 1.44 (1.36–1.52)
	Myocardial infarction	Observational, data mining	HR 1.16 (1.09–1.24)^b^
	Community-acquired pneumonia	Systematic review/meta-analysis	OR 1.49 (1.16–1.92)^b^

Table adapted from Kia et al. [14]

^a^The risk of hypomagnesemia increases to OR 7.22 (1.69–30.83) in patients on concurrent loop diuretics

^b^Risk ratio based on observational study; no association found in RCTs

*HR* hazard ratio, *OR* odds ratio

## Conclusions

In summary, Scarpignato et al. [3] should be commended for this remarkable effort. In addition to the major points detailed above, there are numerous pearls pertinent to comparative PPI pharmacology and metabolism contained within the text. However, the overwhelming message is that the problem with PPIs is that they are good, very good. Consequently, there are a lot of valid indications for their use. However, clinicians cannot be complacent, thinking that the overwhelming efficacy of the drugs in the treatment of peptic esophagitis and ulcer disease will be matched in all other putative applications. Decisions to start, properly dose, continue, or discontinue PPI therapy should be personalized based on indication, effectiveness, patient preferences, and risk assessment. Guidelines are important, but they should not be inflexible. Clinicians need flexibility to tailor therapy to specific patient circumstances and experience. Only then does one achieve the optimal balance between risk and benefit.

## Abbreviations

FDA: Food and drug administration
GERD: gastroesophageal reflux disease
PPI: proton pump inhibitor

## References

[1] Everhart JE, Ruhl CE (2009). Burden of digestive diseases in the United States part I: overall and upper gastrointestinal diseases. Gastroenterology.

[2] Howden CW, Kahrilas PJ (2010). Editorial: just how "difficult" is it to withdraw PPI treatment?. Am J Gastroenterol.

[3] Scarpignato C, Gatta L, Zullo A (2016). Effective and safe proton pump inhibitor therapy in acid-related diseases - A position paper addressing benefits and potential harms of acid suppression. BMC Med.

[4] Qadeer MA, Phillips CO, Lopez AR (2006). Proton pump inhibitor therapy for suspected GERD-related chronic laryngitis: a meta-analysis of randomized controlled trials. Am J Gastroenterol.

[5] Naik RD, Vaezi MF (2013). Extra-esophageal manifestations of GERD: who responds to GERD therapy?. Curr Gastroenterol Rep.

[6] Kieboom BC, Kiefte-de Jong JC, Eijgelsheim M (2015). Proton pump inhibitors and hypomagnesemia in the general population: a population-based cohort study. Am J Kidney Dis.

[7] Lazarus B, Chen Y, Wilson FP (2016). Proton pump inhibitor use and the risk of chronic kidney disease. JAMA Intern Med.

[8] Jalving M, Koornstra JJ, Wesseling J (2006). Increased risk of fundic gland polyps during long-term proton pump inhibitor therapy. Aliment Pharmacol Ther.

[9] Lam JR, Schneider JL, Zhao W (2013). Proton pump inhibitor and histamine 2 receptor antagonist use and vitamin B12 deficiency. JAMA.

[10] Lo WK, Chan WW (2013). Proton pump inhibitor use and the risk of small intestinal bacterial overgrowth: a meta-analysis. Clin Gastroenterol Hepatol.

[11] Xu HB, Wang HD, Li CH (2015). Proton pump inhibitor use and risk of spontaneous bacterial peritonitis in cirrhotic patients: a systematic review and meta-analysis. Genet Mol Res.

[12] Tsai CF, Chen MH, Wang YP (2017). Proton pump inhibitors increase risk for hepatic encephalopathy in patients with cirrhosis in a population study. Gastroenterology.

[13] Lam JR, Schneider JL, Quesenberry CP, et al. Proton pump inhibitor and histamine-2 receptor antagonist use and iron deficiency. Gastroenterology. 2016. doi:10.1053/j.gastro.2016.11.023. Ahead of print. 10.1053/j.gastro.2016.11.023 27890768

[14] Kia L, Kahrilas PJ (2016). Therapy: risks associated with chronic PPI use - signal or noise?. Nat Rev Gastroenterol Hepatol.

